# Global Changes in Gene Expression and Splicing in Alcoholic Liver Disease

**DOI:** 10.21203/rs.3.rs-7380649/v1

**Published:** 2025-09-26

**Authors:** Ilya O. Blokhin, Derek Van Booven, Josepmaria Argemi, Mengying Li, J. Sunil Rao, Estelle Barbier, Markus Heilig, Jin Cheng, Antoine Douaihy, Ramon Bataller, Claes Wahlestedt

**Affiliations:** Harvard University; University of Miami; University of Pittsburgh; University of Miami; University of Miami; Linkoping University; Linkoping University; Pittsburgh Veteran Affairs Medical Center; University of Pittsburgh; University of Pittsburgh; University of Miami

**Keywords:** alcohol use disorder, alcoholic liver disease, splicing, spliceosome, long non-coding RNA, hepatocellular carcinoma

## Abstract

Alcohol use disorder is a widespread illness commonly leading to alcoholic liver disease (ALD) and cirrhosis with an increased incidence of hepatocellular carcinoma (HCC), but the mechanisms of alcohol-related oncogenesis in the liver are incompletely understood. We tested the hypothesis that ALD predisposes to HCC via dysregulation of splicing. RNA sequencing was performed on liver biopsies from patients with different stages of ALD: early alcoholic steatohepatitis (eASH), non-severe alcoholic hepatitis (nsAH), and severe alcoholic hepatitis (sAH); furthermore, explants were collected from patients who underwent liver transplantation due to sAH (exAH). We found that alcohol caused widespread changes in transcriptome in all stages of ALD: among ~ 58,000 analyzed genomic features, ~ 4,900 were altered in eASH, ~ 9,100 – in nsAH, 14,100 – in sAH, and ~ 14,300 – in exAH. We observed thousands of missplicing events in all hepatic conditions, with mutually exclusive exons (MEE) being the most common event and exon skipping (ES) – second most common event. Analysis of ~ 600,000 exons revealed that ALD is associated with a genome-wide effect on exon expression, with ~ 50,000 exons being differentially expressed in eASH, ~ 130,000 – in nsAH, ~ 150,000 – in sAH, and ~ 120,000 – in exAH. To determine whether alcohol directly perturbs splicing, we subjected rats to alcohol vapor for 7 weeks and found that the expression of multiple snRNAs was drastically decreased, while expression of splicing factors was not affected. Screening of oncogenes and tumor suppressors, commonly involved in HCC pathogenesis, revealed that ALD affected the hepatic expression and/or splicing of most of these cancer-related genes. In summary, it appears that alcohol causes profound genome-wide changes in gene expression and splicing in the liver, likely via affecting the spliceosome. This results in altered expression and missplicing of key oncogenes and tumor suppressors involved in HCC, suggesting a novel mechanism of oncogenesis in the liver of patients with ALD.

## Introduction

Alcohol use disorder (AUD) is a chronic condition characterized by a problematic pattern of alcohol use leading to clinically significant impairment. According to the World Health Organization, alcohol consumption accounts for 4.7% of all deaths worldwide^1^. In the United States, 14% of adults currently meet criteria of AUD, 29% – met AUD criteria once during their lifetime^2^; in addition, prevalence of AUD is increasing^3–5^. Annual cost of AUD and alcohol-related disorders is ~$250 billion^6,7^. Due to high prevalence and lack of efficient treatment modalities, AUD causes significant socioeconomic burden. Apart from direct physiological and psychological problems associated with AUD, the burden is connected to alcohol-related medical conditions. One of the major and most common co-morbidities is alcoholic liver disease (ALD) which, in turn, is associated with hepatocellular carcinoma (HCC).

ALD is a broad diagnosis which encompasses the continuum of alcoholic steatosis, alcoholic steatohepatitis (eASH), alcoholic hepatitis (AH), and, finally, cirrhosis^8^. Most patients develop HCC at the stage of cirrhosis, but 11–31% of HCC patients present with HCC in the absence of cirrhotic changes^9,10^. HCC is the most common liver cancer and one of the most aggressive cancers. Worldwide, ~ 850,000 people are diagnosed with HCC every year^11,12^. Therapeutic approaches for advanced stage HCC are very few. Modest improvement was observed after administration of multiple tyrosine kinase inhibitors sorafenib and lenvatinib^13,14^ and immunotherapies^15,16^, but prognosis for patients with advanced HCC is still poor, with an overall survival of around 1 year with the best treatment implemented^17^. HCC remains the second leading cause of cancer-related death in the world claiming ~ 700,000 deaths annually^18,19^. The link between AUD and HCC is well established in case-control studies^20^ and meta-analyses^21^, and alcohol-related HCC was shown to have significantly worse prognosis than HCC related to viral hepatitides^22,23^.

That treatment of HCC is often insufficient may result from an incomplete insight on how HCC arises and progresses. HCC represents a heterogeneous group of tumors with molecular signatures closely connected to risk factors^24^. It is possible that AUD-related HCC develops via distinct molecular mechanism^25^ and that some alcohol-induced molecular changes causing or predisposing to HCC occur at the stage of AH^9,10^. Recent data indicate that potential mechanistic overlap between AUD and HCC may involve impaired splicing. Splicing is a nuclear process of removing introns from pre-mRNA after which mature mRNA is produced and exported into the cytoplasm for translation. Splicing is mediated by a major spliceosome, a nuclear machinery which consists of 5 small nuclear RNAs (snRNA1 (snU1), snU2, snU4, snU5, and snU6) and dozens of splicing factors^26^. Among snRNAs, snU1 and snU2 are considered to be critical. snU1 recognizes 5’ splice site forming the early splicing complex while snU2 recognizes 3’ splice site. Among chief splicing factors with an established link to cancers are SF1, SF3B1, SRSF1, SRSF2, SRSF3, ZRSR2, and U2AF1^27–29^. SF1 is responsible for the recognition of the branch point^30^. When branch point pairs with snU2, SF1 is displaced and branching site is contacted by SF3B1, a core component of the U2 small nuclear ribonucleoprotein (U2 snRNP), which ensures stabilization of U2 snRNP on pre-mRNA^26^. SRSF1, SRSF2, and SRSF3 belong to the family of serine-arginine (SR) splicing factors which contain a protein domain composed of SR repeats; SRSF proteins are recruited with snU1 by RNA polymerase II and function mainly to couple splicing with transcription^31^. ZRSR2 and U2AF1 are required for the recognition of 3’ splice site^32,33^. Regulation of splicing is poorly understood, but there is some evidence indicating that long non-coding RNAs (lncRNAs) might be involved. LncRNAs are non-coding RNA molecules > 200 nucleotides in length which are capable of interacting with both short RNAs and proteins^34^ and thus may serve as a “screwdriver” for spliceosome. It was shown that lncRNA Gomafu (also known as myocardial infarction-associated transcript (MIAT)) affects formation of spliceosomes and inhibits splicing factor SF1^35^. Another lncRNA, MALAT1, interacts with SR splicing factors causing deregulation of splicing in a genome-wide fashion^36,37^.

Data on splicing in AUD are relatively scanty. Alcohol intake was shown to be associated with a missplicing of specific transcripts such as mRNA for AMPA receptors^38^ and GABA-B receptors^39^. Disruption of splicing on a broader scale was observed in the brain cortex of human fetuses exposed to alcohol^40^. In our previous work, we observed AUD-induced genome-wide missplicing in different brain regions^41^. In HCC, splicing is studied more extensively. Analysis of HCC transcriptome identified > 2,000 genes with altered splicing patterns which were distinct in HBV-induced, HCV-induced, and non-viral HCC^42^. It was also shown that HCC was accompanied by 34,163 alternative splicing events in 8,985 genes, and some missplicing patterns were associated with overall survival^43^. However, the extent and mechanisms of missplicing in AUD and ALD-related HCC remain unknown.

The goal of the current study was to determine if AUD may relate to HCC via missplicing in the liver. We found that chronic alcohol use causes genome-wide changes in hepatic gene expression and splicing. Mechanistically, it appears to be mediated by defects in the spliceosomal machinery as well as by an aberrant expression of lncRNAs related to splicing. Numerous oncogenes and tumor suppressors were among affected genes in all alcohol-related hepatic conditions suggesting a novel mechanism contributing to the development of AUD-related HCC.

## Results

### Subjects

Demographics and pertinent clinical data are listed in Table 1. Patients with alcohol-induced liver damage did not differ in age or gender. Child-Pugh, MELD, and ABIC scores all increased from nsAH to sAH, but there was no difference between sAH and exAH groups. Frequency of symptoms of hepatic decompensation such as ascites, hepatic encephalopathy, upper gastrointestinal bleeding, acute kidney injury, and infections tended to increase from nsAH to sAH. White blood cell counts increased and hemoglobin and platelet counts decreased throughout eASH-nsAH-sAH-exAH continuum. As expected, levels of bilirubin alkaline phosphatase were higher in patients with alcohol-induced liver damage. Liver diseases were also associated with low albumin, hyponatremia, and increased prothrombin time (expressed as International Normalized Ratio (INR)). Of note, creatinine levels were normal across phenotypes, ruling out the presence of hepatorenal syndrome.

### Effect of alcohol on hepatic transcriptome

**Figure 1:**
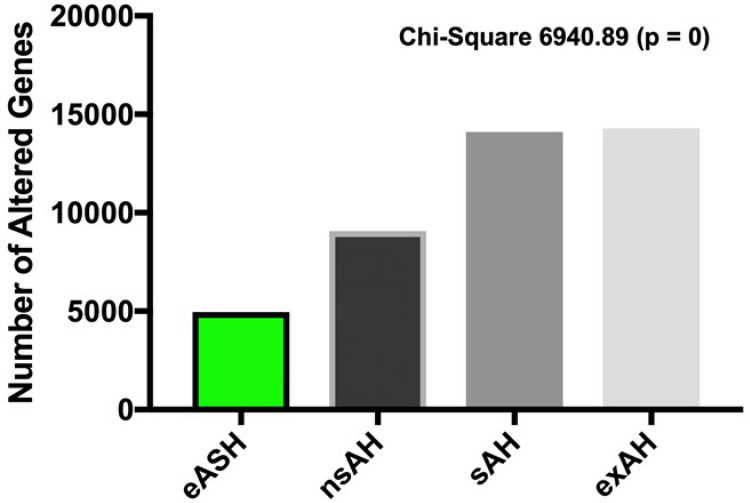
Differentially expressed genes in the liver from patients with alcohol-related hepatic diseases. eASH – early alcoholic steatohepatitis, nsAH – non-severe alcoholic hepatitis, sAH – severe alcoholic hepatitis, exAH – explants from patients who underwent hepatic transplantation due to liver failure.

We have previously shown that there are extensive transcriptomic changes imparted by some stages of ALD^44^, but the assessment of ALD-related transcriptomic reprogramming encompassed only protein-coding genes and did not include the analysis of splice variants. We therefore decided to interrogate RNA sequences datasets in order to evaluate the extent of differential expression and missplicing of both coding and non-coding features. The analysis of expression of 57,820 features confirmed that alcohol caused genome-wide changes in hepatic transcriptome, with a number of differentially expressed genes positively correlating with a disease severity. In eASH, 4,928 were altered (8.5%), in nsAH – 9,075 (15.7%), in sAH – 14,100 (24.4%), and in exAH – 14,292 (24.7%) (Fig. 1). When considering the RNA biotypes, the composition of altered transcriptomes was relatively similar in all alcohol-related condition, with a majority of affected genes (60–70%) being represented by protein-coding genes (Supplementary Fig. S1). Altered transcriptome profiles demonstrated a considerable overlap (data not shown) suggesting that alcohol-related transcriptomic changes are persistent, cumulative along the progression of ALD, and unlikely to be random. To address functional implications of altered transcriptomes, we analyzed transcriptomic overlaps between eASH and later stages of alcohol-induced hepatic damage such as sAH and exAH. Gene Ontology analysis of overlapping suppressed genes revealed that downregulated features are mainly involved in oncogenesis: affected pathways included DNA replication, DNA damage response, cell cycle, and regulation of mitosis (Supplementary Tables 1 and 2).

**Figure 2:**
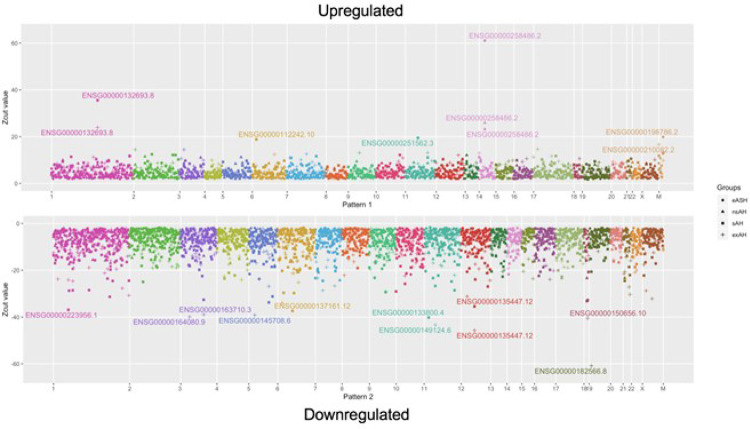
Genes affected in all groups. Top portion of the plot represents 440 transcripts upregulated across all groups, bottom part – 825 downregulated transcripts. X axis signifies chromosomes, y axis corresponds to Zcuts values for each gene that match to four groups. eASH – early alcoholic steatohepatitis, nsAH – non-severe alcoholic hepatitis, sAH – severe alcoholic hepatitis, exAH – explants from patients who underwent hepatic transplantation due to liver failure.

Next, we asked if there are transcriptional changes that persist across all stages of alcohol-induced hepatic damage and assessed determined genes up- or downregulated in all four groups. In total, expression of 1,265 were either increased or decreased throughout eASH-nsAH-sAH-exAH continuum (Fig. 2). 440 genes were upregulated in all groups, 825 – downregulated in all groups (Supplementary Table 3) indicating the broad character of alcohol-induced transcriptomic changes across all conditions.

### Effect of alcohol on splicing

**Figure 3:**
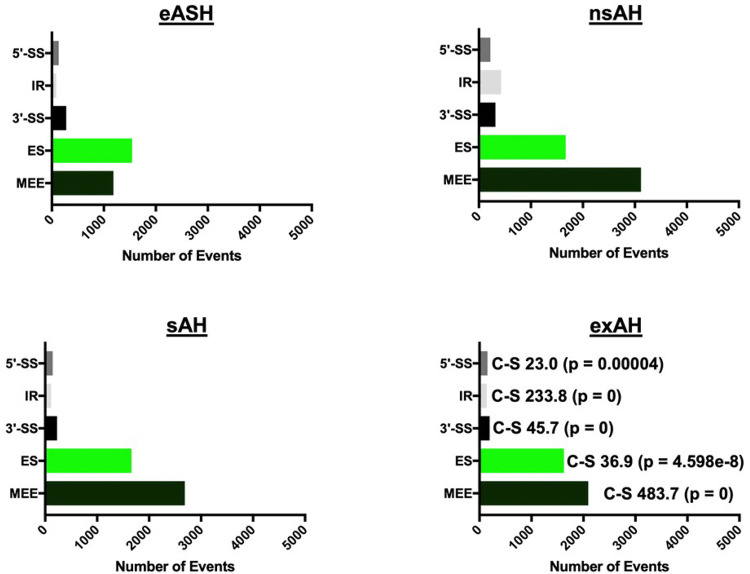
Missplicing patterns in the liver in patients across alcohol-related liver disease spectrum. 5’-SS – alternative 5’ splice site, IR – intron retention, 3’-SS – alternative 3’ splice site, ES – exon skipping, MEE – mutually exclusive exons; eASH – early alcoholic steatohepatitis, nsAH – non-severe alcoholic hepatitis, sAH – severe alcoholic hepatitis, exAH – explants from patients who underwent hepatic transplantation due to liver failure. C-S – chi-square test.

Effect of alcohol on splicing in the liver has not been well studied, and extent and characteristics of hepatic missplicing in ALD are not known. We employed MATS program^45^ to unbiasedly detect such missplicing events as 5’ alternative splice sites (5’-SS), 3’ alternative splice sites (3’-SS), intron retention (IR), exon skipping (ES) and mutually exclusive exons (MEE). In all alcohol-related conditions, ES and MEE were the most common events (Fig. 3). Number of ES was relatively similar across all conditions: 1,542, 1,668, 1660, and 1,623 in eASH, nsAH, sAH, and exAH, respectively. MEE, on the other hand, differed significantly in various conditions. The number of MEEs was 1,186 in eASH, rose to 3,119 in nsAH, and then subsided to 2,668 and 2,094 in sAH and exAH, respectively. Frequency of 5’-SS, 3’-SS, and IR was much lower in comparison to ES and MEE, and the dynamics of their quantitative changes was similar, being observed at the stage of eASH, occurring most frequently at the stage of nsAH, and returning to eASH levels at sAH and exAH stages.

### Effect of alcohol on exon expression

**Figure 4:**
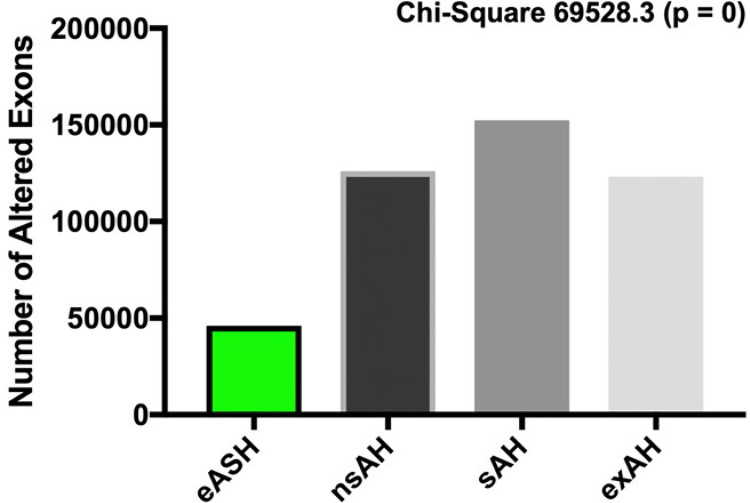
Exon expression in the liver of patients with alcohol-related hepatic diseases. eASH – early alcoholic steatohepatitis, nsAH – non-severe alcoholic hepatitis, sAH – severe alcoholic hepatitis, exAH – explants from patients who underwent hepatic transplantation due to liver failure. Difference in proportion of affected exons between groups was calculated using chi-square test.

Based on the high number of alcohol-induced missplicing events across all conditions, we hypothesized that the variety of differentially expressed transcripts in the liver may be larger than detected on standard differential expression analysis (Fig. 1). We reasoned that missplicing may cause changes in the expression and presence of specific exons without altering a total expression counts of a specific gene, as quantified by conventional pipelines. In such a case, the quantification of individual exons might therefore provide a more accurate insight into alcohol-induced changes in transcriptional landscape and give clues of potentially relevant changes in mRNA and amino acid sequence of key molecular drivers. We used DEXSeq R package to perform exon expression analysis. The interrogation of 588,998 exons showed that alcohol causes genome-wide changes in exon expression, with number of affected exons positively correlating with the stage of disease. Thus, 46,045 (7.8%) exons were altered in eASH, 126,145 (21.4%) – in nsAH, 152,335 (25.9%) – in sAH, and 123,282 (20.9%) – in exAH (Fig. 4).

### Effect of alcohol on snRNAs

**Figure 5:**
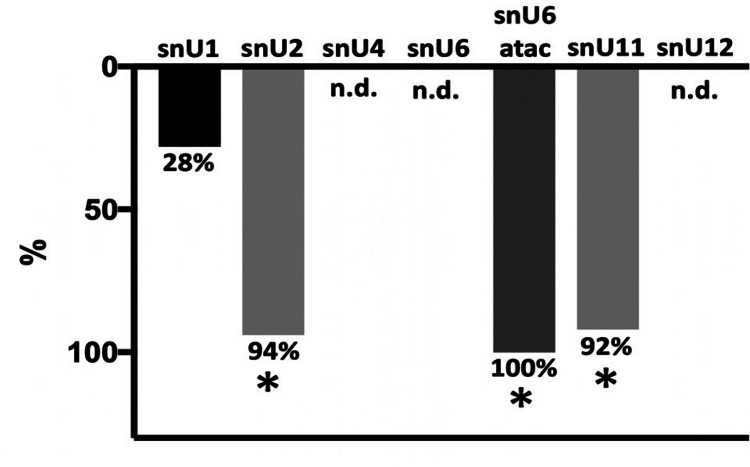
Expression of snRNAs in the liver of rats exposed to alcohol vapor. n.d. – not detected. * – P < 0.05.

We next asked which mechanism may be responsible for such broad alcohol-induced alterations of splicing landscape. Splicing is governed mainly by a major spliceosome, a nuclear machinery which consists of 5 small nuclear RNAs (snRNA) and multiple proteins, known as splicing factors. The snRNAs are involved in the recognition of introns, formation of splicing complexes, and splicing reactions^46,47^. The major spliceosome includes 5 snRNAs: snU1, snU2, snU4, snU5, and snU6. Although data on AUD-induced changes in splicing began to accumulate^40,41,48^, the impact of alcohol on snRNA has not been explored. In most publicly available RNA sequencing datasets in alcohol-related disease studies, snRNAs are not well represented. Only in one of them^49^ did we find robust expression values for most snRNAs; this study showed that treatment of primary hepatocytes with alcohol resulted in decreased expression of snU1 and snU4. That snRNAomes from most public RNA sequencing sources (such as NCBI or TCGA) are missing or unreliable may be due to the length of snRNAs: mature snRNAs are relatively short (< 200 nucleotides) and may not be detected by standard RNA sequencing protocols. Therefore, to investigate the direct effect of alcohol on the snRNAome *in vivo*, we treated rats with alcohol vapor or left untreated for 7 weeks and measured the expression of snRNAs in the liver by real-time polymerase chain reaction (RT-PCR) using custom-made primers. Blood alcohol concentrations (BACs) levels were three times higher in exposed animals (Supplementary Fig. 2). Importantly, the expression of snU2 was 94% downregulated and snU1 was not changed; snU4 and snU6 were not detected (Fig. 5). We also studied the components of the minor spliceosome which is responsible for splicing of atypical snU12-type introns. This specific type of introns constitutes ~ 0.5% of all introns in human genome, and their removal by minor spliceosome is known as non-canonical splicing^50,51^. In the minor spliceosome, the expression of snU6atac was completely abolished, and expression of snU11 was 92% reduced; snU12 was not detected (Fig. 5). Since in our model alcohol was delivered in the form of vapor, we reasoned that similar changes in snRNA expression may be also observed in the lung. We found that snU2 and snU11, but not snU6-atac, were significantly downregulated in the lung of rats subjected to alcohol vapor (Supplementary Fig. 3), although changes were less drastic than in the liver, indicating that the intensity or mode of alcohol-mediated alteration of the spliceosome could be organ-specific.

### Effect of alcohol on splicing factors

**Figure 6:**
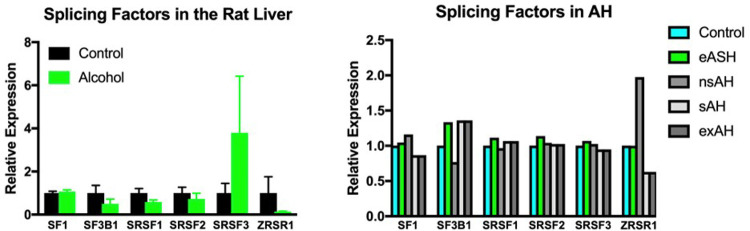
Expression of cancer-related splicing-factors in the liver of rats exposed to alcohol vapor and in the liver of humans with various stages of alcohol-induced hepatic damage. eASH – early alcoholic steatohepatitis, nsAH – non-severe alcoholic hepatitis, sAH – severe alcoholic hepatitis, exAH – explants from patients who underwent hepatic transplantation due to liver failure.

Next, we addressed if alcohol affects expression of splicing factors. Among the main splicing factors with an established link to cancers are SF1, SF3B1, SRSF1, SRSF2, SRSF3, U2AF1, and ZRSR2^27,28^. Analysis of their expression showed no differences between control rats and rats treated with alcohol both in the liver (Fig. 6) and the lungs (Supplementary Fig. 4). To determine if these factors were changed in patients with alcohol-related hepatic conditions, we retrieved and analyzed RNA sequencing data from patients with eASH, nsAH, sAH, and exAH and again found no difference between groups (Fig. 6). It appears that in contrast to snRNAs which are markedly decreased by alcohol, cancer-related splicing factors remain unaltered.

**Figure 7:**
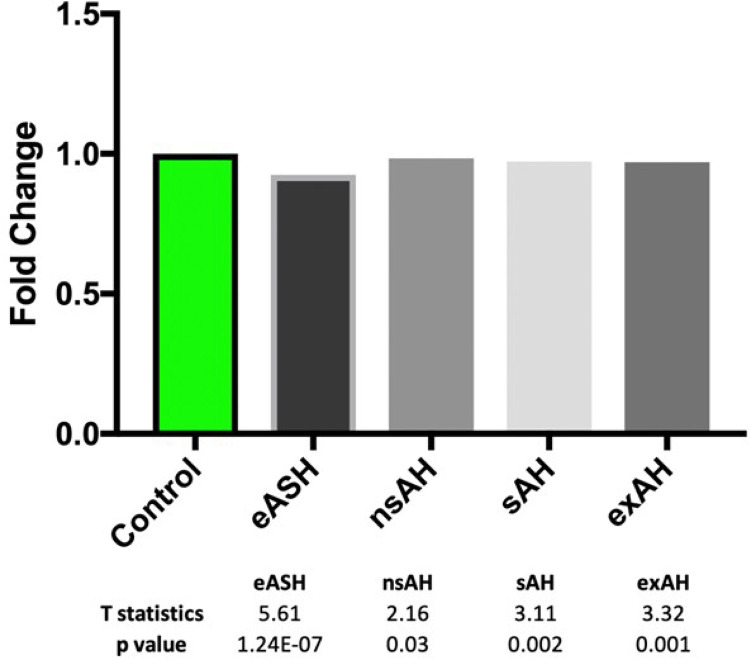
Expression of 127 splicing factors in the liver of patients with various stages of alcohol-induced hepatic damage. eASH – early alcoholic steatohepatitis, nsAH – non-severe alcoholic hepatitis, sAH – severe alcoholic hepatitis, exAH – explants from patients who underwent hepatic transplantation due to liver failure.

Further, since we observed that many of these factors tended to decrease liver and lung of rats exposed to alcohol (Fig. 6 and Supplementary Fig. 4, P > 0.05), we reasoned that an effort to address the functional status of spliceosome by analyzing only 6 splicing factors may be inaccurate, as it may not fully reflect upon how the whole spliceosome may be affected. We compiled the list of all spliceosomal factors using Gene Set Enrichment Analysis (GSEA) (Supplementary Table 4) and checked if alcohol has a concerted effect on spliceosomal proteome. Although there was a statistically significant reduction in global expression of spliceosomal mRNAome, the magnitude of decrease was only in the range 1–7% (Fig. 7) suggesting that protein components of spliceosome are unlikely to be considerably affected by alcohol.

### Effect of alcohol on splicing-related lncRNAs

We observed substantial changes in hepatic lncRNAs in patients with ALD, which correlated with the severity of liver damage. In eASH, 1,108 lncRNAs were differentially expressed, in nsAH – 1854, in sAH – 3,349, and in exAH – 3,353 (Supplementary Fig. 5). Furthermore, the proportion of altered lncRNAs biotypes was different between early and late stages of ALD: while in early stages the pseudogenes were most commonly affected, in non-severe and severe AH stages there was an increased involvement of long intergenic non-coding RNAs and antisense lncRNAs (Supplementary Fig. 5).

We then aimed at analyzing if these alterations were also present in alcohol-related HCC. The analysis of gene expression profiles from TCGA showed that patients with HCC + AUD had 514 differentially-expressed lncRNAs in the liver. The comparison of 3,349 lncRNAs altered in sAH with 514 lncRNAs altered in HCC + AUD demonstrated that 101 were common in these two conditions (Supplementary Fig. 6). Finally, a lncRNA ontology revealed that these lncRNAs are functionally related to splicing, as 3 out of 5 top pathways were represented by splicing and RNA processing (Table 2).

### Effect of alcohol on oncogenes

To address to what extent alcohol-induced changes in the liver affect oncogenes and tumor suppressors, we aligned 14,100 genes altered in sAH with the list of 803 oncogenes from Oncogene Database^52^. 414 were differentially expressed in both conditions (data not shown) suggesting that alcohol-induced transcriptomic changes in the liver posttranscriptionally affect large portion of all cancer-related genes. At the same time, since different cancers are accompanied by specific oncogenes^53,54^, we thought that we should undertake the analysis of oncogenes specific for HCC. In the past few years, extensive efforts by TCGA revealed genes mutated in specific cancers^24,55,56^, and 26 genes were most commonly mutated in HCC^24^. Screened of these genes using our computational pipeline showed that 7 of them were differentially expressed and/or misspliced in eASH, 14 – in nsAH, 17 – in sAH, and 13 – in exAH (Chi-square test = 8.12, p = 0.04, Table 3).

Next, we asked if such a dose-dependent effect is limited to cancer-related genes. We compiled and analyzed the list of 26 random genes which included at least one gene from each chromosome. The similar pattern of increase was found: 5 genes were affected in eASH, 8 – nsAH, 9 – in sAH, and 12 – exAH (Chi-square = 4.37, p = 0.22, Supplementary Table 5). Furthermore, we analyzed 26 genes implicated in spermatogenesis (as a pathway unrelated to the liver) and 26 genes involved in gluconeogenesis (as a pathway related to the liver). For both pathways, we once again observed a similar pattern condition-dependent increase: in eASH-nsAH-sAH-exAH continuum, 6-13-13-14 genes were affected in spermatogenesis (Chi-square = 13.91, p = 0.003, Supplementary Table 6) and 7-14-16-14 – in gluconeogenesis (Chi-square = 7.19, p = 0.06, Supplementary Table 7). We reasoned, therefore, that degree of damage is unrelated to the pathway and rather mirrors the number of genes affected in a specific alcohol-related hepatic disease. We set out to merge and count all differentially expressed and misspliced genes in every alcohol-induced liver condition. Results demonstrated that number of affected genes indeed increases with the severity of hepatic damage: 11.8% genes were affected in eASH, 20.1% – in nsAH, 29.8 – in sAH, and 27.7% – in exAH (p = 0, Supplementary Fig. 7).

Next, we asked if alcohol use may impart broader transcriptomic changes in HCC and compared gene expression profiles from HCC samples between patients with and without AUD. Analysis showed that patients with HCC + AUD have 2-fold increase in a number of differentially increased genes in comparison to patients with HCC alone (Supplementary Fig. 8).

## Discussion

The salient findings of the current study are: 1) ALD is accompanied by pervasive changes in hepatic transcriptomes; 2) ALD is accompanied by genome-wide changes in splicing and expression of exons; 3) Alcohol suppresses hepatic expression of snRNAs of major and minor spliceosomes; 4) Alcohol does not affect expression of splicing factors; 5) Alcohol affects lncRNAs related to splicing, and 6) Alcohol posttranscriptionally affects genes involved in HCC.

In the past few years, alcohol-induced changes in gene expression were relatively well studied in the brain. For example, gene expression profiling on the prefrontal cortex identified 129 altered genes in patients with AUD^57^. It was shown that alcohol-induced changes in the brain are dependent on a cellular lineage, as RNA sequencing demonstrated unique transcriptomic signatures in murine astrocytes^58^ or microglial cells^59^. Furthermore, it was demonstrated in mice that alcohol exposure alters expression of microRNAs (miRs) in prefrontal cortex, nucleus accumbens, and amygdala and that affected miRomes matched expression profiles of protein-coding genes in corresponding regions^60^. In the current study, we show that alcohol causes genome-wide changes in the liver, and these changes appear to be far more pervasive than those in the brain as indicated by our own data from different brain regions^41^ as well as by results from previous studies^57–59^. As of now, it is not known how alcohol may affect gene expression in the liver. Besides splicing, which was the focus of the current study, there are other molecular mechanisms that might be involved. For example, alcohol is associated with aberrant patterns of DNA methylation of CpG islands^61,62^ and changes in histone code^63^; as mentioned, alcohol is also capable of changing the expression of miRs which fine-tune the transcriptome in the cytoplasm^60^. Of note, as extent of transcriptomic damage appears to be distinctly different in the liver and brain, it is possible that mechanism of alcohol-induced changes may be organ-specific and should likely be studied separately across specific tissues.

Splicing takes a central place in a cellular biology: constitutive splicing is responsible for a removal of introns, and alternative splicing generates a proteomic diversity. We observed that in addition to changes in gene expression, alcohol caused multiple missplicing events. There have been very few studies on the effect of alcohol on splicing, and initial investigations mainly focused on missplicing of specific genes. Thus, alcohol was shown to be associated with a missplicing of AMPA receptors^38^ and GABA-B receptors^39^. Ethanol also affected alternative splicing of DRD2 (dopamine D2 receptor) in the pituitary^64^. Later, it was established that alcohol impacts splicing on a broader scale. Kawasawa et al.^40^ found 382 alternative splicing events in the brain cortex of human fetuses exposed to alcohol. Qualitatively, 8 missplicing events were detected: 5’-SS, 3’-SS, MEE, IR, cassette exon, coordinate cassette exon, alternative first exon, and alternative last exon (ES was likely not detectable by employed computational pipeline), with IR being the most frequent event. In our previous work, we observed that AUD caused a genome-wide missplicing in different brain regions, most commonly – ES and MEE^41^. In the current study, analysis of hepatic transcriptomes revealed that ES and MEE were again the major alcohol-induced events. These results are different from the missplicing patterns of another alcohol-related cancer, HNSCC, which also was associated with genome-wide changes in splicing, but alternative splice sites (5’-SS and 3’-SS) and canonical exon skipping were the most common missplicing events^65^. It appears that mechanism, extent, and landscape of alcohol-induced missplicing may differ from one tissue to another.

Scattered data indicate that aberrant splicing in HCC may be a result of a damaged spliceosome; for example, HCC is frequently associated with mutations in splicing factor SF3B1^24,66^. The effect of alcohol on spliceosome has not been well studied, but indirect evidence suggests that the link between AUD and spliceosomal activity may exist. It was shown that vulnerability of neuroprogenitor cells to toxic effects of alcohol was related to the expression of genes related to spliceosome^67^. It was also shown that exposure of neurons to alcohol reduced the expression of SRSF1^68^. Likewise, in murine placenta and embryos, alcohol affected several spliceosomal genes including SRSF1^69^. In our study, we did not see differential expression in cancer-related splicing factors (SF1, SF3B1, SRSF1, SRSF2, SRSF3, U2AF1, and ZRSR2); neither did we see any change in the expression of all components of the spliceosomal proteome. Instead, we observed that alcohol imparted a marked decrease of snRNAs. That snRNAs were affected and splicing factors were not and it was still induced broad missplicing events suggests a primary role of snRNAs in splicing. It is difficult to address if suppression of snRNAs is the only mechanism of missplicing in AUD. Such an experiment would likely require the “snRNAome rescue” experiment, and a precise normalization snRNAome may be technically hard to achieve.

HCC has the second highest worldwide cancer mortality rate, and therapeutic options for metastatic HCC are very limited and modestly efficacious. Many drugs failed to demonstrate efficacy in phase III^70^, likely due to incomplete understanding of mechanisms of HCC in AUD. One explanation of such a poor insight may be a traditional focus on DNA mutations as a driving force of carcinogenesis. Many studies question such an approach. TCGA work on HCC showed that many patients without p53 mutation have inactive functional p53^24^ suggesting that oncogenes may be affected via posttranscriptional mechanism, including splicing. HCC was previously shown to be accompanied by a dysregulated expression of splice isoforms of various genes including DNMT3b, TP53, KLF6^71^, ketohexokinase^72^, and hepcidin^73^. It is possible that AUD-induced HCC represents a separate entity different from, for example, HCC associated with hepatitis C. Such a scenario was shown for esophageal squamous cell carcinoma in which genome sequencing identified six mutational signatures, with one of them specifically linked to alcohol intake^74^. Clearly, other genetic and epigenetic mechanisms are implicated and should be evaluated. For example, it was found that impaired functional status of p53 protein in patients with HCC and non-mutated TP53 could at least partly be attributed to high levels of MDM4, a p53 inhibitor^24^. One could speculate that each of tumor suppressors (oncogenes) involved in HCC may have protein inhibitors (activators). Also, it was shown that alcohol affects such processes related to gene-to-protein flow as RNA transport, ribosomes, and protein processing in the endoplasmic reticulum^67^, and it might be of interest to assess a relative contribution of these mechanisms in processing of HCC-related genes.

In the current study, patients with eASH, nsAH, sAH, and exAH did not differ in terms of alcohol use which is in line with previous findings demonstrating the lack of correlation between alcohol intake and incidence/severity of AH^75,76^. Why are transcriptional and splicing landscapes so different in patients with similar alcohol intake? One may speculate that it is likely related to a genetic predisposition to missplicing. Since splicing is regulated by spliceosome, culprit(s) should be either snRNAs and/or splicing factors. We observed that splicing factors were not affected in all groups. We did not analyze snRNAome in human RNA sequencing datasets, but we cannot rule out the possibility that mutations in genomic snRNAs genes may be involved, and this would warrant a further investigation. It is of interest that both spliceosomes were affected in mice exposed to alcohol suggesting a common mechanism by which AUD may downregulate spliceosomes. Such a mechanism may involve transcription factor(s) orchestrating transcription of snRNAs. Another, posttranscriptional, mechanism of regulation may be represented by lncRNAs. Spliceosome represents a gigantic machinery consisting of snRNAs and splicing factors, and it is functionally coupled with gene expression^77^. Since lncRNAs can interact with DNA, RNA, and proteins, it is possible that they serve as master regulator of spliceosome. In our work, distribution of altered lncRNAomes differed between groups, with pseudogenes dominating early phase of alcohol-induced hepatic damage and lincRNAs and antisense lncRNA – later phases. Some evidence indicates that even individual lncRNAs may cause changes in splicing. Thus, lncRNA MALAT1, an established oncogene activated in many cancers including HCC^78^ and also upregulated in the brain of alcoholics^79^, affected splicing factors HNRNPF and HNRNPF1 as well as levels of serine-arginine-rich splicing factors and affected alternative splicing of hundreds of transcripts^80^. Alternative splicing is globally regulated by sno-lncRNAs, lncRNA flanked by small nucleolar RNA (snoRNAs)^81^ which are a family of conserved nuclear RNAs located in Cajal bodies or nucleoli and participating in snRNAs modifications. Several lncRNAs are able to interact with specific splicing factors^82^. New exciting field is represented by circular RNAs (circRNAs), a covalently closed single-stranded RNA complexes arising from back-splicing. These complexes are highly stable, and it is speculated that they might be capable of competing with pre-mRNA for spliceosome^83^. We interrogated lncRNAs and found that lncRNAs affected in AUD and HCC + AUD were functionally related to splicing. The link between lncRNAs and spliceosomes is currently unknown. One possibility is that lncRNAs may function as “snRNAs sponges”, as a similar function was described for circRNAs which are capable of “sponging” miRs^84^.

**Figure 8:**
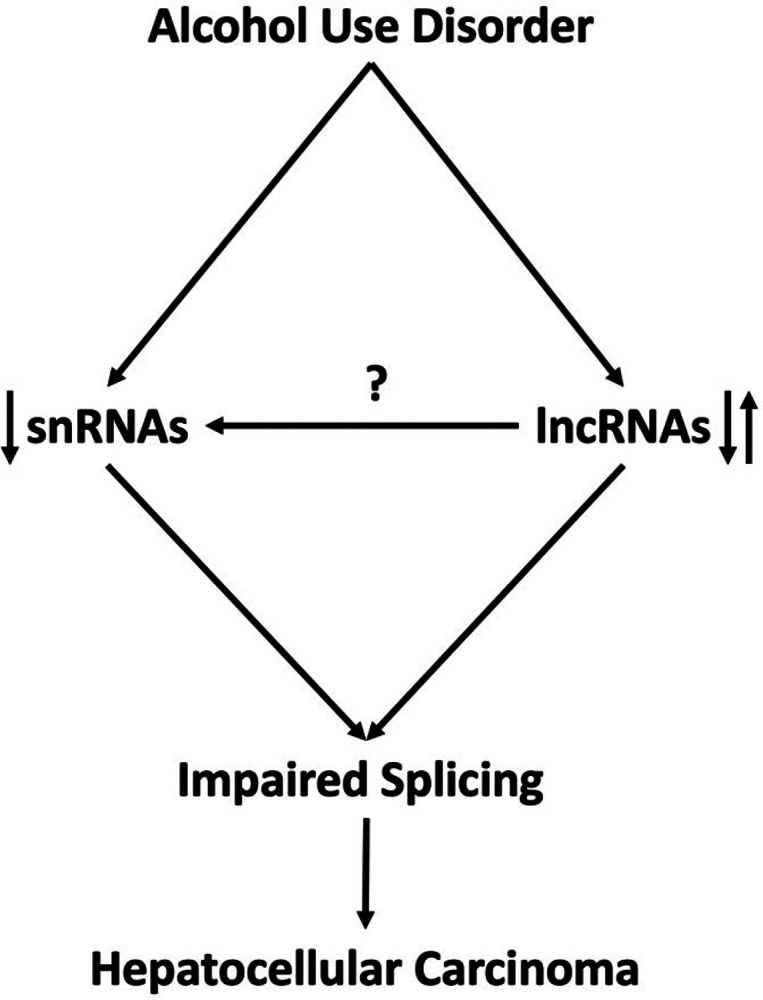
Alcohol use disorder results in missplicing and aberrant gene expression in the liver affecting genes involved in the development of hepatocellular carcinoma. Potential mechanism of missplicing involved suppression of snRNAs and deregulation of lncRNAs.

Our tentative model is shown in Fig. 8. We showed that alcohol perturbs gene expression and splicing in genome-wide fashion across all alcohol-related hepatic diseases. Mechanistically, missplicing appears to be mediated by suppression of snRNAs of major and minor spliceosome. ALD was also associated with altered expression of lncRNAs, but the link between lncRNAs and spliceosome is to be elucidated. Among affected genes are oncogenes and tumor suppressors implicated in the development and progression of HCC.

## Materials and Methods

### Patients

All experiments have been conducted in accordance with the guidelines and regulations of Ethics Committees and Institutional Review Boards of the University of Miami and Linkoping University. RNA sequencing files from liver samples were obtained from the multicentric NIH-funded InTEAM Consortium and are available in the Database of Genotypes and Phenotypes (dbGAP, phs001807)^44^. Patients with non-severe (nsAH) and severe alcoholic hepatitis (sAH) as well as patients with liver failure who underwent liver transplantation were enrolled in the Consortium centers in Europe and United States. Samples from patients with early alcoholic steatohepatitis (eASH) were obtained from Cliniques Universitaires Saint-Luc (Brussels, Belgium). In total, 62 patients were included in this study. Written informed consent was obtained from all patients, and research protocols were approved by the local Ethics Committees and by the central Institutional Review Board of the University of North Carolina at Chapel Hill. Patients were selected according to different clinically relevant stage groups: patients with eASH who were non-obese with high alcohol intake and presented with a mild elevation of transaminases and histologic criteria of steatohepatitis (n = 12); patients with nsAH and sAH who were biopsied before any treatment (n = 11 for nsAH and n = 18 for sAH); and explants from patients with AH who underwent early transplantation due to a liver failure following a well-defined protocol^85^ (exAH, n = 11). Samples from these groups were compared with fragments of non-diseased human livers (n = 10). Patients with malignancies were excluded from the study.

### Sequencing of RNA from liver samples

Sequencing was performed using Illumina HiSeq2000 platform, as previously described^44^. Briefly, libraries were built using Illumina TruSeq Stranded Total RNA Ribo-Zero GOLD (Illumina). Sequencing was paired and multiplexed. 62 paired-end sequenced samples obtained an average of 36,9 million total reads.

### Analysis of Transcriptome

Alignment was performed with the STAR aligner (v.2.5.2a) against the hg19 human genome. Adapters were trimmed by TrimGalore. Raw counts were processed by the Bioconductor R package edgeR and normalized using the TMM method to produce CPM values. Differential expression was calculated using a negative binomial model, and a False Discovery Ratio (FDR) cutoff of < 0.05 was used to define statistical significance.

Next, we analyzed consistent transcriptomic changes across all groups using the Bayesian ANOVA for microarrays (BAM) methodology developed by our group^86^ which employs a specific Bayesian hierarchical model oriented towards adaptive shrinkage. By using model averaging, BAM-provided gene effect estimates are shrunken relative to the standard least square estimates. In this process, primarily the non-differentially expressing gene effects are shrunken. Such a selective shrinkage enabled BAM to optimally balance the total false detections (total number of genes falsely identified as being differentially expressed) against total false non-detections (the total number of genes falsely identified as being non-differentially expressed). This model enabled BAM to use data across all genes and all experimental groups to accurately estimate different levels of sparsity and then to selectively shrink gene effects based on the estimated complexities. BAM estimated gene differential effects (Zcut values) represented the products of posterior inference from the BAM hierarchical Bayesian model. Shrinkage plots were used to identify differentially expressed genes. Significant genes were grouped by differential expression pattern type.

### Gene Ontology of protein-coding genes

Functional analysis of altered transcripts was performed by Gene Ontology Consortium, as described elsewhere^87,88^. Hierarchy of implicated Gene Ontology terms was constructed using default criteria set by developers.

### Analysis of missplicing events

Alignment was performed with the STAR aligner (v.2.5.2a) against the hg19 human genome. Resulting bam files from the STAR alignment were indexed with samtools for use by rMATS, as described elsewhere^45^. Briefly, rMATS pipeline used RNA sequencing reads which were mapped to different splice variants to estimate the isoform proportion, and a hierarchical framework was employed to simultaneously account for estimation uncertainty in individual replicates and variability among replicates. FDR cutoff < 0.05 was used to define statistical significance.

### Exon expression

Analysis of the expression of exons was performed as described elsewhere^89^. Alignment was performed with the STAR aligner (v.2.5.2a) against the hg19 human genome. Adapters were trimmed by TrimGalore. Raw counts were obtained from the dexseq_count.py script contained in the DEXSeq R package (v.1.20.2) on the reference annotation prepared by the dexseq_prepare_annotation.py script in the same package against the GENCODE reference file (v.19). These counts were then fed into DEXSeq to determine exon-specific differential splicing events. Figures were generated using the plotDEXSeq function contained in the DEXSeq package. FDR cutoff < 0.05 was used to define statistical significance.

### Rat model of alcohol use disorder based on vapor chamber

Experiments on animals have been approved by the Institutional Review Board of the Linkoping University and have been carried out in full accord with relevant guidelines and regulations. All methods are reported in accordance with ARRIVE guidelines and regulations^90^. Rats were placed in a vapor chamber with a normal air for one week for a habituation to a new environment (two rats per cage). Then alcohol vapor exposure was slowly increased in the course of 1 week until blood alcohol concentration (BAC) reached ~ 200 mg/dL. Rats were exposed to alcohol vapor for 7 weeks (5 days a week, 14 hours/day) or left unexposed (n = 12 in each group). BAC was measured once per week from one rat per cage (rats were alternating every other week). One week after the last alcohol exposure, animals were sacrificed, and liver and lung samples were collected.

### RNA isolation

RNA was isolated from liver and lung by Qiagen RNeasy kit, in accordance to manufacturer’s instructions. Prior to RNA isolation, samples were incubated in RNAlater-ICE (Thermo Fisher Scientific) overnight at −20°C. DNase treatment was performed using RNase-Free DNase Set (Qiagen). RNA quality and concentrations were determined by Nanodrop 2000, and samples with RNA Integrity Number (A260:A280 ratio) between 1.9–2.1 were included in the study. RNA samples were kept at −80°C until analyzed.

### Quantitative real-time PCR (qRT-PCR)

RNA was reversely transcribed using High Capacity cDNA Reverse Transcription Kit (Thermo Fisher Scientific); reactions were run on a Veriti 96 Well Fast Thermal Cycler (Applied Biosystems); cycling conditions were 25°C for 10 minutes, 37°C for 2 hours, and 85°C for 5 minutes. For real-time PCR, cDNA was mixed with a master mix consisting of TaqMan Universal PCR Master Mix (Roche), nuclease-free water, and 20X or 40 X TaqMan primers. qRT-PCR was performed on QuantStudio 6 Flex (Thermo Fisher Scientific) using probe-based assays from Integrated DNA Technologies. Cycling conditions were 50°C for 2 minutes, 95°C for 10 minutes, and 40 cycles of 95°C for 15 seconds and 60°C for 1 minute. Data were analyzed using the comparative threshold cycle (ΔΔCT) method, as described previously^91^, normalized to β-actin, and expressed relatively to a control group. Primers for protein-coding genes were purchased from a pre-made stock of Integrated DNA Technologies. Primers for human and rat snRNAs were designed either by ourselves or by Integrated DNA Technologies, and their sequences are listed in Supplementary Table 8.

### Computational Analysis of Coding and Non-coding Features

The Cancer Genome Atlas (TCGA, www.cancer.gov/tcga) was used to retrieve RNA sequencing files from patients with HCC and HCC + AUD. For most patients with HCC, alcohol intake was well documented on TCGA; however, since DSM-5-defined criteria of AUD were not included in TCGA social history, we arbitrarily defined AUD group as individuals consuming 4 or more drinks a day at least 4 days a week; control group included subjects with no alcohol intake. Other inclusion criterion was being alive at the time of cancerous tissue harvest (to avoid possible RNA degradation in postmortem samples). 10 patients were included in each group. All datasets incorporated in the analysis were generated by aligning reads to GRCh38 using STAR; genes were annotated using GENCODE v.22, and their expression was presented in a FPKM-UQ format.

Gene Set Enrichment Analysis database^92^ was used to obtain the list of spliceosomal proteins and to create the lists of proteins involved in gluconeogenesis and spermatogenesis as well as the list of 26 random genes which included at least 1 gene from each chromosome. List of cancer-related genes was retrieved form the Oncogene Database^52^, a literature-based curated database of oncogenes and tumor suppressors. Functional status of altered lncRNAs of interest was studied using “LncRNA Ontology” database^93^ which employs the pipeline based on transcriptional and epigenetic profiles of lncRNAs and protein-coding genes; default criteria set by developers were used to execute the ontology.

### Statistical Analysis

Statistical analysis between two groups was performed using two-tailed Student’s *t* test. Data are expressed as mean ± SEM unless otherwise stated. Proportions in events in different groups were assessed using chi-square test; rejection of the null hypothesis (that proportions are the same across groups) indicated that proportion is different in at least one group. Difference between expression of splicing factors was determined using the Test for Fold Change of Two Means. Differences with *P* < 0.05 were considered to be statistically significant.

## Supplementary Material

Supplementary Files

This is a list of supplementary files associated with this preprint. Click to download.

- SupplementaryTableS2.OverlappingDownregulatedTranscriptomeineASHandexAH.xlsx
- SupplementaryTableS4.ListofSpliceosomalGenes.xlsx
- SupplementaryTableS6.GenesInvolvedinSpermatogenesis.xlsx
- SupplementaryTableS1.OverlappingDownregulatedTranscriptomeineASHandsAH.xlsx
- SupplementaryTableS5.GenesfromDifferentChromosomes.xlsx
- SupplementaryTableS7.GenesInvolvedinGluconeogenesis.xlsx
- SupplementaryTableS3.ConsistentTranscriptomicChanges.xlsx
- SupplementaryTableS8.snRNAsprimers.xlsx
- SupplementaryFiguresS1S8.docx

## Figures and Tables

**Table 1.** Baseline characteristics of patients and controls at the time of liver biopsy

	Normal livers n=10	Early ASH n=12	Non-Severe AH n=11	Severe AH n=18	Severe AH (explants) n=11	ANOVA F-Statistic (p-value) or Chi-square Test
**Demographics**						
Age - median (IQR)	32(29.0–49.0)	52(48.8–58.2)	46 (42.5–49.5)	51(47.2–57.8)	45(41.0–52.0)	4.56(0.003)
Gender - male n (%)	7(70)	7(58.3)	7 (63.6)	11(61.1)	7(63.6)	0.353(0.986)
**Severity scores** – median (IQR)						
Child-Pugh	N/A	N/A	7 (6–8.5)	11(9–11.8)	12(11.5–13)	34.55(0.000)
MELD	N/A	N/A	12 (10.5–14.5)	24(22–27.8)	26.1(24.2–31.9)	26.22(0.000)
ABIC	N/A	N/A	6 (5.5–6.4)	8.3(7.9–8.8)	8.8(8–9.5)	7.642(0.002)
**Decompensations** – n (%)						
Ascites	0	0	5 (45.5)	13(72.2)	7(77.8)	2.927(0.232)
Hepatic Encephalopathy	0	0	0(0)	5(27.8)	1(11.1)	4.156(0.125)
Upper GI Bleeding	0	0	1(9.1)	1(5.6)	2(22.2)	1.804(0.406)
Acute Kidney Injury	0	0	2 (18.2)	9 (50)	N/D	1.74(0.187)
Infections	0	0	1 (9.1)	6(33.3)	5(55.6)	4.995(0.082)
**Lab parameters** - median (IQR)						
Hemoglobin g/dL	14.6(13.2–15.3)	14.3(13.7–16.1)	11.8(9.8–13.3)	11.5 (11.0–12.7)	N/D	7.914(0.000)
WBC ×109/L	5.7(5.2–7.1)	5.6(4.7–6.9)	6.7 (5.3–7.7)	9.6 (8.1–14.3)	10.2(9.6–13.2)	5.749(0.001)
Platelets ×109/L	237(217–267.5)	189.5(131.8–236.2)	166(104–208.5)	115 (82.2–183.5)	N/D	6.659 (0.001)
AST (U/L)	21.5(19.2–25.8)	107(66–142)	104(51–196.5)	133 (117.5–218.2)	118(104–127.8)	7.207(0.000)
ALT (U/L)	25(16.8–31.2)	70.0(58.5–89.2)	36(30.5–45)	55 (35–65.5)	N/D	8.252(0.000)
Bilirubin mg/dL	0.6(0.5–0.7)	1.2 (0.8–1.4)	1.7(0.9–3.2)	19 (12.9–26.2)	19.5(15.1–27.4)	40.02(0.000)
GGT (U/L)	17(14.0–23.2)	388(208.2–674.5)	709(274.5–1202.5)	293.5(175.5–456.8)	N/D	5.014(0.004)
ALP (U/L)	147(112.5–184.8)	89.5(70.0–117.2)	393(249.5–531)	402(300.5–494.8)	N/D	15.66(0.000)
Albumin (g/dL)	4.6(4.4–4.6)	4.5(4.2–4.7)	3.2(2.9–3.7)	2.5 (2.3–3.0)	2.3(2.2–2.7)	43.34(0.000)
Creatinine mg/dL	0.84(0.76–0.89)	0.6(0.59–0.73)	0.69(0.60–0.86)	1.02(0.73–1.31)	0.77(0.60–1.02)	1.743(0.154)
Sodium (mEq/L)	140.0(139.2–141.0)	139.5(137.5–140.0)	137(135–138)	132.0(130.2–136.5)	N/D	7.721(0.000)
INR	1.02(0.99–1.05)	0.99(0.94–1.02)	1.25(1.15–1.43)	1.67 (1.56–1.81)	2.05(1.90–2.80)	23.67(0.000)

ASH: alcoholic steatohepatitis; AH – alcoholic hepatitis; IQR: interquartile range; WBC: while blood cells; AST: aspartate aminotransferase; ALT: alannine aminotransferase; GGT: gamma-glutamyl transpeptidase; ALP: alkaline phosphatase; INR: international normalized ratio. N/A: not applicable; N/D: not determined (or missing data).

**Table 2.** LncRNA Ontology of LncRNAs Altered in sAH and HCC+AUD

Biological Process	Number of lncRNAs
mRNA splicing, via spliceosome	23
DNA repair	21
RNA processing	19
mRNA processing	18
DNA metabolic process	17

**Table 3.** Oncogenes Affected in the Liver of Patients with Various Stages of Alcohol-induced Damage

Transcript	eASH	nsAH	sAH	exAH
TP53		**x**	**x**	**x**
CTNNB1	**x**	**x**	**x**	
ALB	**x**	**x**	**x**	**x**
AXIN1		**x**	**x**	**x**
BAP1	**x**		**x**	
KEAP1				
NFE2L2				
LZTR1				
RB1		**x**	**x**	**x**
PIK3CA				
RPS6KA3		**x**	**x**	**x**
AZIN1			**x**	**x**
KRAS		**x**	**x**	**x**
IL6ST	**x**	**x**	**x**	**x**
RP1L1				**x**
CDKN2A		**x**	**x**	**x**
EEF1A1		**x**	**x**	
ARID2				
ARID1A	**x**	**x**	**x**	**x**
GPATCH4				
ACVR2A				
APOB	**x**	**x**	**x**	**x**
CREB3L3	**x**	**x**	**x**	**x**
NRAS			**x**	
AHCTF1		**x**	**x**	
HIST1H1C				
	7	14	17	13

## Data Availability

RNA sequencing files from liver samples were obtained from the multicentric NIH-funded InTEAM Consortium and are available in the Database of Genotypes and Phenotypes (dbGAP, phs001807).
